# Schon gewusst …?

**DOI:** 10.1007/s41975-023-00288-w

**Published:** 2023-03-13

**Authors:** Petra Stute

**Affiliations:** Abteilung für Gynäkologische Endokrinologie und Reproduktionsmedizin, Universitätsklinik für Frauenheilkunde, Inselspital Bern, Friedbühlstrasse 19, 3010 Bern, Schweiz

## Einfluss einer HRT auf das Risiko für eine Depression

### Originalpublikation

Wium-Andersen MK et al (2022) Association of hormone therapy with depression during menopause in a cohort of Danish women. JAMA Netw Open 5(11):e2239491.

### Hintergrund.

Erst im August 2022 wies das Positionspapier der Nordamerikanischen Menopause Gesellschaft (NAMS) [1] daraufhin, dass (1) die antidepressive Wirkung von Östrogenen ähnlich stark ist wie die von Antidepressiva, wenn sie depressiven Frauen in der Perimenopause mit oder ohne Hitzewallungen verabreicht werden (Level II) und (2) transdermales Östradiol mit sequentiellem, mikronisiertem Progesteron depressive Symptome bei euthymischen Frauen in der Perimenopause vorbeugen kann (Level II). Diese Statements werden nun von einer prospektiven Kohortenstudie (Level IV) in Frage gestellt.

### Zusammenfassung.

In einer dänischen, registerbasierten, prospektiven Kohortenstudie wurden alle Frauen, die im Zeitraum 1995–2017 45 Jahre alt wurden, eingeschlossen (*n* = 825.238). Das Follow-up endete 2018 und war somit für die eingeschlossenen Frauen unterschiedlich lang (ca. 1–23 Jahre). Während des Follow-up wurden 13.069 Frauen (1,6 %) aufgrund einer Depression hospitalisiert (Inzidenz 17,3 Fälle pro 10.000 Personenjahre). Während des Follow-up initiierten 189.821 Frauen (23 %) eine systemische (Östrogen mono (ET), Östrogen-Gestagen-Kombination (EPT) oder lokale Hormonersatztherapie (HRT)). Das mediane Alter bei HRT-Start war 55 Jahre. Im Median wurden 33 HRT-Verschreibungen eingelöst, wobei ca. 2/3 der Frauen mindestens fünf Verschreibungen einlösten, am häufigsten für eine lokale HRT (lokal 65,8 % vs. ET 7,8 % vs. EPT 26,4 %). Die Prävalenz einer früheren Depression war insgesamt gering (2,4 %), wobei HRT-Anwenderinnen jedoch seltener davon betroffen waren als Nicht-Anwenderinnen (1,2 % vs. 2,8 %). Die Prävalenz einer früheren Anwendung von Antidepressiva (19 %) und Schlafmedikamenten (14,4 %) war jedoch deutlich höher! Das Ziel der Studie war es, die Assoziation zwischen einer HRT und einer späteren Depressionsdiagnose zu untersuchen. Die Assoziationen wurden unter Verwendung von Cox-Proportional-Hazards- und Fixed-Effects-Poisson-Regressionsmodellen untersucht. Alle Hazard Ratios (HR) wurden für Bildungsniveau, Ehestatus, Geburten, frühere Anwendung von hormonalen Kontrazeptiva und Schlafmedikamenten (aber nicht Antidepressiva!) und Komorbiditäten (Diabetes, Bluthochdruck, Apoplex, Herzerkrankung, frühere Depression) adjustiert. Nur eine systemische, nicht aber eine lokale HRT war mit einem erhöhten Risiko für eine hospitalisierungspflichtige Depression verbunden; und dies v. a. bei 48- bis 50-jährigen HRT-Starterinnen (HR 1,50, 95 % KI 1,24–1,81) sowie v. a. im 1. Jahr nach Beginn einer Behandlung mit ET (HR 2,03, 95 % KI 1,21–3,41) bzw. EPT (HR 2,01, 95 % KI 1,26–3,21). Die Fallzahlen waren allerdings recht klein: Von den 649.279 45-jährigen Frauen ohne Depression in der Vorgeschichte wurden innerhalb des 1. Beobachtungsjahr 74 Frauen aufgrund einer Depression hospitalisiert (vs. 534 der Nicht-Anwenderinnen). Eine lokale HRT nach 54 Jahren war dagegen mit einem signifikant geringeren Depressionsrisiko verbunden. Die Autoren kommen zu dem Schluss, dass eine systemische HRT mit einem höheren Depressionsrisiko verbunden ist, insbesondere in den Jahren unmittelbar nach Behandlungsbeginn, während eine lokal verabreichte Hormonbehandlung mit einem geringeren Depressionsrisiko für Frauen ab 54 Jahren verbunden ist.

## Kommentar

Die Skandinavischen Registerstudien bestechen regelmässig durch ihre hohen Fallzahlen. Dennoch lohnt sich ein kritischer Blick. Das Durchschnittsalter bei HRT-Start lag in der Postmenopause, was sich auch in der bevorzugt lokalen HRT-Verschreibung widerspiegelt. Die Indikation für die Verschreibung einer systemischen HRT ist unklar, was bei Registerstudien auch nicht anders zu erwarten ist. Allerdings heisst es im Ergebnisteil, dass dies die erste Beobachtungsstudie sei, die Frauen prospektiv beobachte, die eine HRT aufgrund einer klinisch diagnostizierten Depression begannen. Wenn das so stimmte, dann würde die Studie eher den Einfluss einer HRT auf das Hospitalisierungsrisiko (also den Schweregrad) einer Depression untersuchen. Wichtig an der Stelle ist, dass der Studienendpunkt die hospitalisierungspflichtige Depression war. Andere, auch schwächere, vielleicht auch positive Affektveränderungen wurden nicht erfasst. Die Prävalenz einer früheren Anwendung von Psychopharmaka ist deutlich höher als die der im Register dokumentierten früheren Depressionen; diese Diskrepanz wird nicht geklärt. Auch wird für die frühere Anwendung von Antidepressiva nicht adjustiert!? Auch zur systemischen HRT bleiben viele Fragen offen: unklar ist, welche Präparate eingesetzt wurden, welche Gestagene, welcher Applikationsmodus (oral, transdermal), welche Applikationsschemata (sequentiell, kontinuierlich-kombiniert), welche Dosis. Ausserdem wird die HRT-Therapiedauer nicht angegeben, da nur in „eingelösten Rezepten“ gerechnet wird. Wie aber ist in Dänemark die Verschreibungseinheit definiert? Wie z. B. in Deutschland mit maximal drei HRT-Packungen pro Rezept? Oder eher wie in der Schweiz, wo vorwiegend Jahresrezepte ausgestellt werden? Oder ganz variabel? Als Fazit bleibt, dass nach wie vor viele Fragen zum Einfluss einer HRT auf die Stimmung offenbleiben. Eine einzelne Studie sollte uns aber nicht davon abhalten, Frauen mit klimakterischem Syndrom die 1st-line Therapie HRT zu verschreiben.

## Brain Fog in den Wechseljahren

### Originalpublikation

Maki PM, Jaff NG (2022) Brain fog in menopause: a health-care professional’s guide for decision-making and counseling on cognition. Climacteric 301–9. 10.1080/13697137.2022.2122792.

### Hintergrund.

Kognitive Beschwerden sind in den Wechseljahren häufig und mit einer reduzierten Lebensqualität verbunden [2]. Spätestens seit der Pandemie aufgrund von SARS-CoV‑2 Infektionen ist den meisten der Begriff „brain fog“ geläufig, der nun auch zunehmend im Kontext der Wechseljahre gebraucht wird. Wie sollten Frauen mit „brain fog“ in den Wechseljahren beraten werden?

### Zusammenfassung.

Unter „Brain Fog“ in den Wechseljahren versteht man verschiedene kognitive Symptome, die sich häufig als Schwierigkeiten im Gedächtnis und in derAufmerksamkeit manifestieren. Diese kognitiven Veränderungen in den Wechseljahren sollten nicht mit einer Demenz verwechselt werden. Eine Demenz vor 64 Jahren ist selten. Zu den häufigsten kognitiven Beschwerden zählen Schwierigkeiten beim Lernen und im verbalen Gedächtnis. Die Symptome beginnen meist während der menopausalen Transition. Die Beschwerden können störend und subjektiv besorgniserregend sein, der normale kognitive Funktionsumfang wird jedoch in der Regel beibehalten; nur etwa 11–13 % der Frauen weisen eine klinisch signifikante Beeinträchtigung auf. Die kognitiven Symptome sind mit Veränderungen der Östrogenserumkonzentration, vasomotorischen Beschwerden, Schlaf und Stimmung assoziiert. Die Behandlung dieser Symptome kann sich positiv auf die Kognition auswirken. Es stellt sich die grundsätzliche Frage nach der Rolle der HRT im Hinblick auf die Kognition und Demenz. Eine HRT wird derzeit international weder zur Behandlung kognitiver Beschwerden in den Wechseljahren noch zur Prävention eines kognitiven Abbaus bzw. Demenzentwicklung empfohlen. Fragen zum Einfluss einer HRT auf die kognitiven Fähigkeiten bei Frauen mit störenden Hitzewallungen oder bei Frauen in der Perimenopause können mangels entsprechender Studien noch nicht beantwortet werden. Bei Frauen mit früher Menopause (< 45 Jahren) unterstützen Östrogene den Erhalt der kognitiven Funktion und reduzieren das Demenzrisiko. Wenn eine HRT in der frühen Postmenopause begonnen wird, ist kein negativer Einfluss auf die Kognition zu erwarten. Gleiches gilt für den Einsatz von reinen Östrogenen in der späten Postmenopause. Bisher konnte nur für die kombinierte HRT mit konjugierten equinen Östrogenen (CEE) und Medroxyprogesteronacetat (MPA) ein negativer Einfluss auf die kognitive Funktion beobachtet werden, wenn diese nach 65 Jahren gestartet (!) wird. Die Kombination von oralem Estradiol und vaginalem Progesteron scheint dagegen auch bei Start in der späten Postmenopause sicher zu sein. Verschiedene modifizierbare Risikofaktoren sind mit der kognitiven Gesundheit assoziiert. Hierzu zählen Adipositas, Bluthochdruck, Diabetes, mangelnde körperliche Aktivität, Rauchen, mangelnde kognitive Aktivität, wenig soziale Interaktion, Schwerhörigkeit und Depression. Diese Risikofaktoren sollten möglichst gut gemanagt/reduziert werden. Es liegen bisher keine ausreichenden Daten vor, um zwischen kognitiven Beschwerden aufgrund der Wechseljahre und aufgrund von Long COVID unterscheiden zu können. Allerdings scheinen exekutive Funktionseinbussen bei SARS-CoV‑2 ein herausragendes Merkmal zu sein, welche in der Regel in den Wechseljahren nicht beobachtet werden.

## Kommentar

Das Review bietet praktische Empfehlungen zum Management von Frauen mit kognitiven Beschwerden in den Wechseljahren. Am wichtigsten ist, dass diese kognitiven Beschwerden nicht ein Vorbote der Demenz darstellen, wie häufig befürchtet. Auch das Demenzrisiko unter HRT wird aufgegriffen und verdeutlicht, dass ein erhöhtes Risiko bisher nur für asymptomatische (!) > 65-jährige (!) Frauen beobachtet wurde, die erst zu diesem Zeitpunkt (!) mit einer oralen kombinierten HRT bestehend aus CEE und MPA (!) beginnen [3].
